# Prevalence of Burnout Syndrome for Public Schoolteachers in the Brazilian Context: A Systematic Review

**DOI:** 10.3390/ijerph18041606

**Published:** 2021-02-08

**Authors:** Natalia P. Montoya, Lia C. O. B. Glaz, Lucas A. Pereira, Irineu Loturco

**Affiliations:** 1 Península Institute, São Paulo 01452-000, Brazil; natalia.puentes@institutopeninsula.org.br (N.P.M.); lia.glaz@institutopeninsula.org.br (L.C.O.B.G.); lucas.pereira@narsp.com.br (L.A.P.); 2 NAR-Nucleus of High Performance in Sport, São Paulo 04753-060, Brazil; 3 Department of Human Movement Sciences, Federal University of São Paulo, São Paulo 11015-020, Brazil; 4 Faculty of Life Sciences and Education, University of South Wales, Pontypridd, Wales CF037 1DL, UK

**Keywords:** emotional exhaustion, psychological disturbs, stressful environments, pedagogue, educator

## Abstract

The aim of this systematic review was to examine the prevalence of burnout-related symptoms in Brazilian schoolteachers who work in public schools. The literature search was conducted using the following databases: PubMed-MEDLINE, Scopus, and Web of Science. Peer-reviewed studies published in English, Spanish, or Portuguese were considered for inclusion. A total of 2106 records were identified through database searching and 7 additional studies were identified through other sources. Based on the inclusion and exclusion criteria, 19 studies were included in the systematic review. Burnout syndrome prevalence was assessed through seven distinct questionnaires. Overall, the majority of the studies presented high methodological quality. Brazilian schoolteachers exhibited high levels of emotional exhaustion (21–69%), high or moderate degrees of depersonalization (8–32%), and high levels of personal realization and enthusiasm toward the job (30–90%). From these data, it is possible to infer that Brazilian schoolteachers are, in general, affected by burnout syndrome. However, and, surprisingly, they seem to be motivated and idealistic, as demonstrated by their high levels of personal realization and enthusiasm toward the job (30–90%). This likely favors the implementation of programs designed to avoid or reduce burnout, deal with stress, and enhance teaching quality.

## 1. Introduction

Burnout syndrome is considered by the World Health Organization as a real and critical risk factor for workers [1], being able to provoke both physical and mental deterioration among different professional groups. Briefly, burnout can be defined as a typical syndrome in the labor environment, which acts as a chronic process in response to excessive occupational stress [2]. This psychological disorder generally occurs when subjects endure prolonged periods of increased stress [3], as a consequence of exposure to substantial amounts of time working or to recurrent (and demanding) tasks and responsibilities.

From an applied perspective, burnout syndrome may be identified through the presence of three problematic components: low sense of work fulfillment, high levels of physical exhaustion, and cynicism [4,5,6]. This syndrome can affect a wide variety of occupations, resulting in absenteeism, lack of job commitment, dissatisfaction, and other job-related issues, which certainly compromise the productivity of various types of organizations and companies [7,8]. For these reasons, several authors have devoted a great deal of effort and attention to examining the prevalence and potential effects of this psychological disorder, as well as its respective sets of symptoms and consequences, on worker productivity [4,7,8,9,10].

In fact, the occurrence of burnout syndrome is commonplace across numerous professions [4,8,9,10]. For example, nurses who are directly caring for patients in hospitals and nursing homes frequently experience higher levels of burnout than those working in pharmaceutical companies, who do not have direct contact with patients or their families [10]. As a consequence, the quality of their work and level of satisfaction with their nursing care are severely compromised. The same holds true for police officers, especially those usually engaged in daily interactions with citizens and in crime-prevention. It has been reported that both fatigue and burnout syndrome may impair the efficient functioning of police organizations, thus generating increased levels of aggressiveness and intolerance among these public service employees [9]. Other professionals who often experience elevated levels of stress during their daily routines are elementary school teachers [11]. Intense workload, lack of autonomy, indiscipline and low sociability of students, lack of support from colleagues, principals, and institutions, and a gradual loss of professional status over the years are only some examples of stressors commonly associated with teaching.

Currently, teaching is considered one of the most stressful occupations among those involving interpersonal relationships [12], especially in problematic contexts such as those commonly found in developing countries. As such, the level of stress in schoolteachers in Brazil tends to be negatively affected by many factors, for example, inadequate working environments with excessive students per class and extensive working hours [6,11,13,14]. In addition, the context of vulnerability in which they work seems to pose a high level of complexity to traditional teaching practices, most commonly related to ensuring the academic development of students [4,5,13]. Factors beyond the control of the teachers, such as the prevalence of violence, food insecurity, lack of family structure, and a quasi-absence of effective systemic support for children and adolescents, impose great responsibility on these professionals [15]. In structured and well-organized contexts, students are usually “more prepared to learn” (in both social and academic aspects), while in more vulnerable contexts, children and adolescents have to frequently deal with conflicting circumstances and demands. These issues certainly affect teachers’ motivation and attitude toward their profession.

In addition to the stressful situations commonly faced by teachers in Brazil, their systematic formation does not adequately prepare these professionals [16]. Several studies indicated that teacher education in Brazil lacks integration with school practices, a problem that persists across their professional life [17,18]. As found in several other countries, there is a preference for a more traditional teacher training approach based on content and curriculum, and the teacher preparation fails to provide these professionals with technical and emotional abilities (e.g., perceiving and regulating emotions and feelings) to effectively cope with adverse and challenging environments [18]. Together, these factors may potentially lead to high levels of stress and, hence, to an increased risk of burnout among Brazilian schoolteachers. In this regard, this systematic review was conducted to examine the prevalence of burnout-related symptoms in samples exclusively composed of Brazilian schoolteachers.

## 2. Methods

### 2.1. Literature Search and Data Resources

This research was completed in accordance with the Preferred Reporting Items for Systematic Review (PRISMA) guidelines [19]. The literature search included studies published until 25th September 2020 and was conducted using the following databases: PubMed MEDLINE, Scopus, and Web of Science. Keywords were defined based on previous investigations [5,6] and the aims of this study by the four authors (N.P.M., L.C.O.B.G., L.A.P., and I.L.). As part of the search strategy, the Boolean operators “AND” and “OR” were used in conjunction with the following keywords: Brazil, Brazilian, Teacher, Educationist, Educator, Instructor, Pedagogue, Tutor, Faculty, Burnout, “Emotional exhaustion”. Reference lists from relevant articles were also examined to find other potentially eligible studies.

### 2.2. Eligibility Criteria

Randomized peer-reviewed studies published in English, Spanish, or Portuguese were considered for inclusion and no age or sex restrictions were imposed. Studies were included based on these criteria: (1) cross-sectional original studies; (2) Brazilian teachers; (3) quantitative assessment of Burnout. In relation to the exclusion criteria, studies were not considered for analysis if they included (1) university professors; (2) private school teachers; (3) teachers from other countries; (4) no quantitative measurement of burnout.

### 2.3. Study Selection

The initial search was carried out by two researchers (L.A.P. and I.L.). After the removal of duplicates, titles and abstracts were screened, and studies not meeting the eligibility criteria were excluded. Subsequently, full texts of the remaining articles were analyzed. Next, in a blind, independent fashion, two authors selected the studies for inclusion (L.A.P. and I.L.), following the eligibility criteria. If no agreement was obtained, a third researcher (N.P.M.) was consulted.

### 2.4. Methodological Quality Assessment

Included studies were assessed for methodological quality by two authors (L.A.P. and I.L.) using the Joanna Briggs Institute Critical Appraisal Checklist for Studies Reporting Prevalence Data [20]. If no agreement was obtained, a third researcher (N.P.M.) was consulted. All studies were included based on the eligibility criteria regardless of the outcome of methodological quality assessment.

### 2.5. Data Extraction

Main outcomes, sample size, and characteristics of the teachers were extracted from the included manuscripts by one author (L.A.P.) and subsequently checked for completeness and accuracy by a second author (I.L.). All required descriptive data were presented in the articles, so no additional contact with the authors was necessary. Any disagreements during the process of data extraction and analysis were resolved by consensus among the four authors (N.P.M., L.C.O.B.G., L.A.P., and I.L.).

## 3. Results

**Figure 1:**
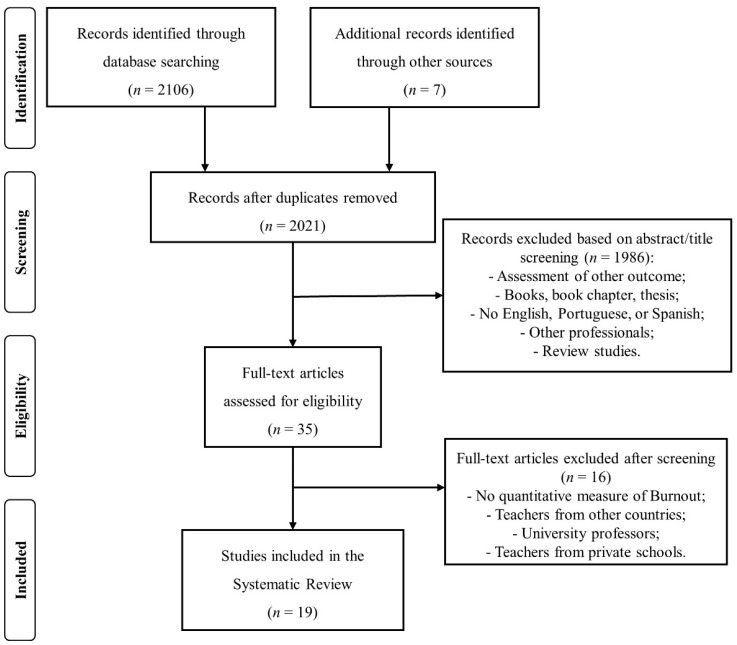
Flow diagram of the process of study selection.

Figure 1 depicts the flow diagram of the process of study selection. A total of 2106 records were identified through database searching and 7 additional studies were obtained through other sources. After removing duplicates, the title and abstract of 2021 studies were screened and 1986 studies were excluded. As a result, 35 studies were assessed for eligibility. After a full text analysis, 16 studies were additionally excluded, and 19 studies were included in the systematic review [2,4,13,14,21,22,23,24,25,26,27,28,29,30,31,32,33,34,35].

The main outcomes, sample size, and characteristics of the teachers of the included studies are shown in Table 1. From the 19 articles included, 7 studies assessed burnout through the complete version of the Maslach Burnout Inventory (MBI) [2,22,23,26,30,34,35], 5 studies used the MBI Educators Survey (MBI-ED) [4,13,28,32,33], and 1 study implemented the MBI Human Services Survey (HSS) [31]. In addition, a further four tools were used to assess burnout syndrome in the teachers, with each questionnaire being implemented in one study as follows: Spanish Burnout Inventory, Education Professionals version (SBI-Ed) [21], Burnout Syndrome Inventory (BSI) [24,29], Burnout Syndrome Evaluation Questionnaire (CESQT) [14,25], and Burnout Teachers Questionnaire (BTQ-R) [27].

A total of 4567 teachers were assessed in the 19 studies included in the systematic review, with the majority of the sample composed of women (~70%). In addition, the sample of the included studies was composed of teachers with very distinct career time, varying from <10 years of experience to >20 years. The total class time, in hours per week, also demonstrated high variation among the teachers assessed, ranging from <20 h to >60 h of weekly class time. In relation to the burnout outcomes, the item of emotional exhaustion demonstrated moderate to high scores. In contrast, teachers demonstrated good scores related to professional realization, which is associated with positive experiences during teaching practice and enthusiasm for work.

Table 2 shows the methodological quality assessment of the 19 included studies. Overall, the majority of the studies presented high methodological quality. From the 19 studies, 4 did not present adequate sample sizes, 9 studies did not clarify if the analysis was conducted with sufficient coverage of the identified sample, and 4 studies did not report if the teachers were assessed in a standardized and reliable way. The other items of the quality assessment checklist were met by all studies.

## 4. Discussion

This systematic review analyzed the existing literature regarding the prevalence of burnout-related symptoms in Brazilian schoolteachers published between the years 2003 and 2020. After examining the results of 19 peer-reviewed studies that met our inclusion criteria, we observed that (1) overall, irrespective of the measurement instrument used (i.e., MBI, MBI-ED, HSS, SBI-Ed, BSI, CESQT, and BTQ-R), Brazilian schoolteachers regularly present high levels of emotional exhaustion (from 21 to 69% prevalence) accompanied by burnout prevalence; (2) in addition, Brazilian schoolteachers frequently report high or moderate degrees of depersonalization (from 8 to 32% prevalence), which also predispose them to develop burnout (from 30 to 70% prevalence); and (3) despite these “negative outcomes”, curiously, these individuals demonstrate high levels of personal realization and enthusiasm toward the job (from 30 to 90% prevalence). From these data, it is possible to infer that Brazilian schoolteachers are, in general, affected by burnout syndrome.

Several articles presented emotional and psychological exhaustion as main outcomes from their analyses [2,4,22,26,28,30,31]. In general, these variables were shown to be related to work overload, negative feedback, and interpersonal conflicts [21]. As a consequence, it is plausible to infer that, as teaching is an interpersonal profession [36], emotional exhaustion and, therefore, burnout may also impact students [26], peers, and families. In addition, it can be expected that burnout affects school environments and, as a result, teaching quality [27]. Another negative effect is the possible relationship between burnout syndrome and voice disorders (e.g., loss of voice, rough voice, dry cough, and pain when speaking) [14], which means that this syndrome may be associated with physical exhaustion and intense physical activities. According to this evidence, for example, older women who teach lots of students are regularly exposed to challenging teaching environments, frequently experiencing heavy workloads, which increases the risk of developing burnout [4]. Other studies highlighted the negative effects of burnout on regular daily activities of schoolteachers, such as driving performance [31] or gathering with family, evidencing that this syndrome also affects teaching safety and life quality. Although some results revealed that burnout perception depends on some specific characteristics of schoolteachers (e.g., age and professional experience), altogether, these alterations will certainly compromise teaching quality [2]. Acknowledging and understanding the complexity of this occupational disorder [29] is essential to create specialized approaches to reduce burnout-related symptoms in schoolteachers and include them in public policy measures for the promotion of personal health status and well-being, for example, specialized meetings and seminars that deal with self-diagnosis, emotional management skills, and time management (i.e., time with family vs. working time) [25,34]. The adoption of multifaceted programs comprising the above-mentioned characteristics, specifically focused on developing these social and psychological qualities, will probably lead to positive effects on mental health, especially in some dimensions that are more flexible than others, such as illusion for work, problem-focused coping, and dealing with emotions at work [25]. From an applied standpoint, strategies to reduce burnout may be suggested as viable alternatives to humanize teaching practice, strengthen interpersonal relationships, and promote mental, physiological, and social health [21,26]. The implementation of these programs in school environments might positively influence teaching behavior and, as a result [27], the quality of education [31].

Other articles also identified indolence and depersonalization as additional negative outcomes of burnout syndrome [4,22,26,28,33]. In fact, negative attitude, indifference, and insensitivity are effects that any professional who develops burnout may feel. In the particular case of schoolteachers, these effects may have substantial impacts on students’ progression since they compromise learning expectations [26] and academic performance. As an example of indolence and lack of expectance, in Brazil, according to SAEB 2017 (i.e., National System of Monitoring and Evaluation for Education), 33% of teachers believe that less than half of their students will enter college [17]. Moreover, in cases with high levels of emotional detachment and dehumanization (i.e., two symptoms related to indolence and depersonalization), depression was strongly and positively correlated with different dimensions of burnout [24]. Therefore, due to these problematic issues, teacher indolence and depersonalization certainly have negative impacts on future generations because of the background of interpersonal risks [21]. Based on these considerations, government agencies and policy makers should stimulate and encourage principals and school staff to create programs to reduce the levels of stress among Brazilian schoolteachers, which, in turn, could lead to decreased levels of emotional detachment and dehumanization.

Despite the lack of social support, excessive workload associated with critical work conditions, and low expectations about their professional performances, surprisingly, in general, Brazilian schoolteachers present high levels of personal realization and enthusiasm toward the job (Table 1) [4,13,21,22,26,34,35]. These positive feelings may increase the success of programs developed to prevent and reduce burnout syndrome and prepare these professionals to deal with stress, which, in turn, could enhance teaching quality [13]. In fact, the presence of burnout among schoolteachers requires the reconstruction and reformulation of many individual and collective attitudes towards the teaching profession, which also requires a solid redefinition of concepts and values in school environments [22]. Nevertheless, the high levels of personal realization and enthusiasm toward the job in Brazilian schoolteachers contrast with the consistent presence of emotional exhaustion and depersonalization but also reveal the intangible strength of teaching, the power of education, and the passion that these professionals have to act as agents of social transformation. These positive behaviors and attitudes toward work will probably facilitate the adherence to programs designed to reduce burnout and improve well-being among schoolteachers, this being another reason for developing these strategies in the Brazilian context.

This review is limited by the heterogeneity among the study designs, especially by the wide assortment of scales used to assess burnout in Brazilian schoolteachers (seven distinct questionnaires), which compromises data interpretation, thus precluding more robust analyses and conclusions. Lastly, it should be recognized that this data collection and analysis per se do not represent substantial progress in this regard, as numerous organizational and work-related problems may potentially compromise the implementation of efficient burnout prevention strategies in the Brazilian scenario.

## 5. Conclusions

There is a crucial need to revisit current research practices and utilize a standardized measurement instrument to assess burnout in these professionals. This will facilitate data comparison and management and, more importantly, the implementation of evidence-based programs developed from multiple studies on burnout. From the gathered and analyzed data, it is possible to state that Brazilian schoolteachers exhibit high levels of emotional exhaustion accompanied by burnout prevalence, regularly presenting high or moderate degrees of depersonalization and indolence. Nonetheless, it is interesting to note that these professionals also possess high levels of personal realization and enthusiasm toward work. Hence, although affected by burnout syndrome, Brazilian schoolteachers seem to be motivated and idealistic, which possibly favors the implementation of programs designed to avoid (or reduce) burnout, deal with stress, and enhance teaching quality.

## Figures and Tables

**Table 1:** Characteristics and main outcomes of the studies included in the systematic review.

Reference	N	Sex		Age	Career Time	Weekly Class Time	Instrument	Main Outcomes
		Women	Men					
Batista et al. (2010)	265	91%	9%	43.5 ± 10.4 years	<10 years (30%) >10 years (70%)	<40 h (32%) >40 h (68%)	MBI-ED	**EE:** 33.6% high, 66.4% low **DP:** 8.3% high, 91.7% low **PR:** 56.6% low, 43.4% high
Carlotto (2011) *	535	-	-	-	-	-	MBI-ED	**EE:** 2.40 ± 0.40 **DP:** 1.56 ± 0.66 **PR:** 2.03 ± 0.74
Carlotto and Câmara (2019)	679	92%	8%	42.0 ± 9.0 years	17.0 ± 8.9 years	34.0 ± 11.6 h (16–57 h)	SBI-Ed	**ETJ:** 89.1% high, 10.9% low **PE:** 15.3% high, 84.7% low **Indolence:** 20.9% high, 79.1% low **Guilt:** 20.8% high, 79.2% low
Costa and Silva (2012)	100	79%	21%	20–60 years	<10 years (32%) 10–15 years (12%) 15–20 years (19%) >20 years (37%)	<20 h (8%) 20–39 h (41%) 40–60 h (51%)	MBI	**EE:** 26.3 ± 13.6 (moderate) **DP:** 6.94 ± 6.28 (moderate) **PR:** 35.6 ± 7.5 (moderate)
Dalcin and Carlotto (2018)	20	100%	-	42.7 ± 10.3 years	18.4 ± 7.6 years	35.5 ± 14.2 h	CESQT	**Illusion:** 2.87 ± 0.91 **Exhaustion:** 1.86 ± 0.93 **Indolence:** 1.27 ± 0.71 **Guilt:** 1.17 ± 0.60
Da Silva et al. (2018)	100	-	-	41.9 ± 9.9 years	1–5 years (19%) >6 years (81%)	<30 h (61%) >30 h (39%)	BSI	**PWC:** 36% problem, 64% no problem **NWC:** 33% problem, 67% no problem **EE:** 37% problem, 63% no problem **EmD:** 40% problem, 60% no problem **DEZ:** 22% problem, 78% no problem **PF:** 11% problem, 89% no problem
Da Silva and Almeida (2011)	20	100%	-	33.6 years	9.3 years		MBI	**EE:** median: 21 (55.5% low) **DP:** median: 5 (96% low) **PR:** median: 31 (73.7% high)
De Brito Mota et al. (2018)	208	77%	23%	41.0 ± 8.8 years	16.1 ± 9.3 years	<20 h (39%) 20–40 h (49%) >40 h (12%)	CESQT	**Illusion:** 88% high, 12% low **Exhaustion:** 30% high, 70% low **Indolence:** 3% high, 97% low **Guilt:** 14% high, 86% low
Koga et al. (2015)	804	67%	33%	<35 years (32%) >35 years (68%)	13.3 ± 9.0 years	28.2 ± 9.3 h	MBI	**High EE:** 22.5% **High DP:** 22.6% **Low PR:** 19.0%
Levy et al. (2009)	77	-	-	<40 years (44%) >40 years (56%)	-	<60 h (44%) >60 h (56%)	BTQ-R	**Burnout Presence:** 70% **Without Burnout:** 30%
Lopes and Pontes (2009)	20	65%	35%	41–50 years	15.3 ± 10.2 years	-	MBI-ED	**EE:** mean: 2.93, variance: 1.38 **DP:** mean: 1.60, variance: 0.85 **PR:** mean: 3.39, variance: 0.94
Lorenzo et al. (2020)	13	100%	-	35.0 ± 7.2 years 25–47 years	8.0 ± 5.5 years	39.4 ± 6.8 h	BSI	**NOC:** 69% presence, 31% absence **POC:** 31% presence, 69% absence **EE:** 69% presence, 31% absence **EmD:** 38% presence, 62% absence **DEZ:** 38% presence, 62% absence **PR:** 77% presence, 23% absence ***Burnout:*** 46% presence, 54% absence
Moreira et al. (2009)	149	56%	44%	24–57 years	<10 years (38%) 10–20 years (30%) >20 years (32%)	<20 h (48%) >20 h (52%)	MBI	**EE:** 37% high, 31% medium, 32% low **DP:** 16% high, 37% medium, 47% low **PR:** 31% high, 52% medium, 17% low
Salvagioni et al. (2020)	509	66%	34%	41.8 ± 9.9 years 19–67 years	-	38.0 ± 11.5 h	MBI-HSS	**EE:** 26.6 ± 8.0 (moderate) **DP:** 10.7 ± 4.1 (moderate) **PR:** 29.4 ± 5.6 (high)
Santana et al. (2012)	85	65%	35%	21–64 years	1–5 years (36%)	18 classes/week (55%)	MBI	**EE:** 47% high, 33% medium, 20% low **DP:** 32% high, 53% medium, 15% low **PR:** 80% high, 19% medium, 1% low
Silva and Carlotto (2003)	61	49%	51%	36.5 years	-	-	MBI-ED	**EE:** W = 3.01 ± 1.24; M = 2.46 ± 1.17 **DP:** W = 0.84 ± 0.82; M = 1.25 ± 0.85 **PR:** W = 4.46 ± 0.94; M = 4.58 ± 0.95
Souza et al. (2016)	220	51%	49%	42.2 ± 11.6 years 22–50 years	16.4 ± 10.6 years 1–45 years	<30 h (25.5%) 31–40 h (33.2%) >41 h (37.7%)	MBI-ED	**EE:** 2.05 ± 0.92 (27% high, 36% medium, 29% low) **DP:** 1.49 ± 0.68 (8% high, 31% medium, 61% low) **PR:** 1.57 ± 0.68 (83% high, 16% medium, 1% low)
Tabeleão et al. (2011)	601	84%	16%	21–68 years	<10 years (35%) 10–20 years (33%) >20 years (32%)	<20 h (62%) >20 h (38%)	MBI	**High EE:** 21% **High DP:** 30% **Low PR:** 14% ***Burnout Prevalence:*** 31%
Tibúrcio and Moreno (2009)	101	72%	28%	40.8 ± 8.6 years	22.6 ± 4.9 years	38.6 ± 14.6 h	MBI	**EE:** 40% high, 27% medium, 33% low **DP:** 20% high, 29% medium, 51% low **PR:** 44% high, 43% medium, 13% low

BSI: Burnout Syndrome Inventory; BTQ-R: Burnout Teachers Questionnaire; CESQT: Burnout Syndrome Evaluation Questionnaire; DEZ: Dehumanization; DP: Depersonalization; ED: Educators Survey; EE: Emotional Exhaustion; EmD: Emotional Detachment; ETJ: Enthusiasm Toward the Job; h: hours; HSS: Human Services Survey; MBI: Maslach Burnout Inventory; NOC: Negative Organizational Conditions; NWC: Negative Work Conditions; PE: Psychological Exhaustion; PF: Personal Fulfillment; POC: Positive Organizational Conditions; PR: Professional Realization; PWC: Positive Work Conditions; SBI-Ed: Spanish Burnout Inventory, Education Professionals Version; * The participants in this study comprised private and public-school teachers, and although the burnout scores were divided between these two categories, their sociodemographic characteristics were not, which is why we did not report it.

**Table 2:** Critical appraisal of the included studies.

Reference	Q1	Q2	Q3	Q4	Q5	Q6	Q7	Q8	Q9	%
Batista et al. (2010)	Y	Y	Y	Y	U	Y	Y	Y	Y	89
Carlotto (2011)	Y	Y	Y	Y	Y	Y	Y	Y	Y	100
Carlotto and Câmara (2019)	Y	Y	Y	Y	Y	Y	Y	Y	Y	100
Costa and Silva (2012)	Y	Y	Y	Y	U	Y	U	Y	Y	78
Dalcin and Carlotto (2018)	Y	Y	N	Y	U	Y	Y	Y	Y	78
Da Silva et al. (2018)	Y	Y	Y	Y	Y	Y	Y	Y	Y	100
Da Silva and Almeida (2011)	Y	Y	N	Y	U	Y	Y	Y	Y	78
De Brito Mota et al. (2018)	Y	Y	Y	Y	U	Y	U	Y	Y	78
Koga et al. (2015)	Y	Y	Y	Y	U	Y	Y	Y	Y	89
Levy et al. (2009)	Y	Y	Y	Y	U	Y	Y	Y	N	78
Lopes and Pontes (2009)	Y	Y	N	Y	Y	Y	Y	Y	Y	89
Lorenzo et al. (2020)	Y	Y	N	Y	Y	Y	Y	Y	Y	89
Moreira et al. (2009)	Y	Y	Y	Y	Y	Y	U	Y	Y	89
Salvagioni et al. (2020)	Y	Y	Y	Y	Y	Y	Y	Y	Y	100
Santana et al. (2012)	Y	Y	Y	Y	U	Y	U	Y	Y	78
Silva and Carlotto (2003)	Y	Y	Y	Y	Y	Y	Y	Y	Y	100
Souza et al. (2016)	Y	Y	Y	Y	U	Y	Y	Y	Y	89
Tabeleão et al. (2011)	Y	Y	Y	Y	Y	Y	Y	Y	Y	100
Tibúrcio and Moreno (2009)	Y	Y	Y	Y	Y	Y	U	Y	Y	89
**Total (%) Yes**	100	100	79	100	53	100	74	100	95	-

Y, yes; U, unclear; N, no. Critical appraisal questions: Q1. Was the design appropriate to address the target population? Q2. Were study participants sampled in an appropriate way? Q3. Was the sample size adequate? Q4. Were the study subjects and the setting described in detail? Q5. Was data analysis conducted with sufficient coverage of the identified sample? Q6. Were valid methods used for identification of the condition? Q7. Was the condition measured in a standard, reliable way for all participants? Q8. Was there appropriate statistical analysis? Q9. Was the response rate adequate, and if not, was the low response rate managed appropriately?
